# Impact of non-pharmaceutical interventions during COVID-19 on future influenza trends in Mainland China

**DOI:** 10.1186/s12879-023-08594-1

**Published:** 2023-09-27

**Authors:** Xiaofan Liu, Ying Peng, Zhe Chen, Fangfang Jiang, Fang Ni, Zhiyong Tang, Xun Yang, Cheng Song, Mingli Yuan, Zhaowu Tao, Junjie Xu, Ying Wang, Qiong Qian, Rob M. Ewing, Ping Yin, Yi Hu, Weihua Wang, Yihua Wang

**Affiliations:** 1 Department of Pulmonary and Critical Care Medicine, Tongji Medical College, The Central Hospital of Wuhan, Huazhong University of Science and Technology, Wuhan, 430014 Hubei China; 2 Wuhan Centers for Disease Control and Prevention, Wuhan, 430024 Hubei China; 3 Biological Sciences, Faculty of Environmental and Life Sciences, University of Southampton, Southampton, SO17 1BJ UK; 4 Department of Biostatistics, University of Iowa, Iowa City, IA 52242 USA; 5 Institute for Life Sciences, University of Southampton, Southampton, SO17 1BJ UK; 6 Department of Epidemiology and Biostatistics, School of Public Health, Tongji Medical College, Huazhong University of Science and Technology, Wuhan, Hubei 430014 China; 7 NIHR Southampton Biomedical Research Centre, University Hospital Southampton, Southampton, SO16 6YD UK

**Keywords:** Non-pharmaceutical interventions, COVID-19, Influenza, Forecast, Time series methods, STL model, SARIMA model, Seasonal decomposition

## Abstract

**Background:**

Influenza is a common illness for its high rates of morbidity and transmission. The implementation of non-pharmaceutical interventions (NPIs) during the COVID-19 pandemic to manage its dissemination could affect the transmission of influenza.

**Methods:**

A retrospective analysis, between 2018 and 2023, was conducted to examine the incidence of influenza virus types A and B among patients in sentinel cities located in North or South China as well as in Wuhan City. For validations, data on the total count of influenza patients from 2018 to 2023 were collected at the Central Hospital of Wuhan, which is not included in the sentinel hospital network. Time series methods were utilized to examine seasonal patterns and to forecast future influenza trends.

**Results:**

Northern and southern cities in China had earlier outbreaks during the NPIs period by about 8 weeks compared to the 2018–2019. The implementation of NPIs significantly reduced the influenza-like illness (ILI) rate and infection durations. Influenza B Victoria and H3N2 were the first circulating strains detected after the relaxation of NPIs, followed by H1N1 across mainland China. The SARIMA model predicted synchronized H1N1 outbreak cycles in North and South China, with H3N2 expected to occur in the summer in southern cities and in the winter in northern cities over the next 3 years. The ILI burden is expected to rise in both North and South China over the next 3 years, with higher ILI% levels in southern cities throughout the year, especially in winter, and in northern cities mainly during winter. In Wuhan City and the Central Hospital of Wuhan, influenza levels are projected to peak in the winter of 2024, with 2 smaller peaks expected during the summer of 2023.

**Conclusions:**

In this study, we report the impact of NPIs on future influenza trends in mainland China. We recommend that local governments encourage vaccination during the transition period between summer and winter to mitigate economic losses and mortality associated with influenza.

## Background

NPIs have been implemented globally to curb the transmission of severe acute respiratory syndrome coronavirus 2 (SARS-CoV-2) [1–3]. NPIs in China to combat the SARS-CoV-2 epidemic have been the longest and most rigorous globally, spanning from January 2020 to January 2023, and included measures such as quarantine, mask-wearing, hand hygiene, social distancing, travel restrictions, online learning, and community lockdowns. The implementation of these measures has resulted in a decrease in the transmission of COVID-19 [4]. Additionally, it has had an impact on the transmission patterns of other viruses that are spread directly, such as influenza, leading to reduced levels of activity during subsequent seasons worldwide [5]. However, the relaxation of NPIs along with increased susceptibility to influenza viruses may result in significantly higher infections and healthcare-seeking rates globally compared to pre-pandemic seasons [6]. For instance, Australia experienced a substantial winter influenza season in May 2022. This serves as a warning that there is a potential for greater influenza activity globally when NPIs are eased, as there has been a decline in influenza immunity [1–5]. Lei and colleagues employed an susceptible-vaccinated-infectious-recovered-susceptible (SVIRS) model to project the potential resurgence of influenza activities following the relaxation of NPIs, suggesting the importance of heightened influenza vaccination rates for effective epidemic control [7].

The World Health Organization (WHO) defines ILI as the presence of fever equal to or greater than 38 °C, accompanied by cough or sore throat, and with onset within the preceding 10 days [8]. Influenza and COVID-19 have similar clinical symptoms and transmission routes. Their activity is carefully monitored in China through sensitive, laboratory-based surveillance systems.

As the COVID-19 epidemic situation improves, China implemented class B notifiable infectious diseases for COVID-19 on 8 January 2023 and relaxed its NPIs accordingly. Subsequently, the Chinese National Influenza Center (CNIC) reported a sharp increase in ILI cases since the sixth week of 2023. This underscores the urgent need to anticipate the progression of future influenza seasons in mainland China and evaluate the potential effects of proactive intervention strategies, such as enhancing influenza vaccination rates. The aim of this study is to forecast potential future outbreaks of influenza strains, including their anticipated magnitude and temporal onset, drawing from the context of prior influenza activity. Moreover, this study seeks to assess the influence of NPIs during the COVID-19 pandemic on the transmissibility of influenza viruses, while also providing projections for the upcoming influenza trends in China.

## Study design and methods

### Data distributions and collection

We analyzed the numbers of ILI cases in 2 geographically diverse areas in mainland China, including northern and southern cities. The *Qin Ling* Mountains and *Yellow* River serve as the geographical divide between North and South China, which is geographically demarcated into 2 distinct and markedly disparate climatic regions, encompassing the southern and northern territories. The influenza virus exhibits varying degrees of adaptability to these divergent climatic conditions, thus prompting the focal investigation of the southern and northern regions of China within this study. Of particular interest is Wuhan, a prototypical southern city that thrives as a central metropolis, hosting a populace numbering in the millions. Renowned as a pivotal transportation nexus, Wuhan’s extensive network of connections spans various directions throughout the country. Consequently, our research designates Wuhan as the primary subject of inquiry. Furthermore, the Central Hospital of Wuhan, a prominent healthcare institution, and recognized for its status as a pre-eminent non-sentinel hospital, serves as an optimal site for the collection of influenza samples.

The coverage of the Chinese National Influenza Surveillance Network spans 32 provinces, including autonomous regions and municipalities, and comprises 410 network laboratories and 554 sentinel hospitals. The sentinel hospitals are responsible for gathering respiratory specimens from patients with ILI and transporting them to the network laboratories for real-time reverse transcription polymerase chain reaction (RT-PCR) testing [9–11]. Positive specimens are then forwarded to the CNIC for additional analysis of the viruses.

In this study, we collected 426 weeks of influenza data from the Chinese Center for Disease Control and Prevention, and weekly counts of ILI patient visits and influenza-positive samples from the CNIC website (https://ivdc.chinacdc.cn/cnic/zyzx/lgzb/; accessed on 3 March 2023). The data spanned from the first week of 2018 to the ninth week of 2023, and all cases were diagnosed in accordance with the “Diagnostic criteria for influenza (WS 285–2008)”. The influenza data included the weekly percentage of patients with ILI and the number of positive cases for 6 types of influenza (A-H1N1, A-H3N2, A-Not-subtyped, B-Not-Determined, B-Victoria, B-Yamagata) in both south and north cities of China. The ILI patient ratio (ILI%) was calculated by dividing the number of ILI patients with the total number of outpatient and emergency cases.

Importantly, to verify the reliability of the data collected from the sentinel hospitals in China, the study also obtained data on confirmed influenza cases at the Central Hospital of Wuhan between 2018 and 2023 (Approval No.: WHZXKYL2023-002). The Central Hospital of Wuhan is a comprehensive hospital that provides care for both pediatric and adult patients and is not part of the sentinel hospital network. A total of 4,185 influenza patients were collected. Influenza population data for Wuhan City were obtained from Hubei Provincial Center for Disease Control and Prevention.

To evaluate the variations observed pre- and post-implementation of NPI for COVID-19, data on ILI were further divided into pre-NPIs (2018–2019) and post-NPIs (2020–2023) seasons.

### Analysis of influenza sequence characteristics using seasonal-trend decomposition using loess (STL)

The study utilized STL [12] to examine the long-term trend, seasonal trend, and random effect of influenza in China from the first week of 2018 to the tenth week of 2023. The following equation was used to analyze the influenza sequence characteristics:

$$Xt=Tt+St+It$$

Here, *Xt* represents the actual value of ILI% at time *t*, while *Tt*, *St*, and *It* correspond to the long-term trend, seasonal trend, and random effects, respectively.

### Seasonal auto-regressive integrated moving average (SARIMA) model to forecast the possible future influenza trends in China

The SARIMA model is a time series model utilized for prediction [13]. It is an extension of the ARIMA model that incorporates a seasonal component to account for periodic fluctuations in the data. The following equation represents the SARIMA model:

$$\begin{array}{ccccc}{Y_t} & = \mu + {\phi _1}({{{Y}}_{t - 1}} - \mu ) + \ldots + {\phi _p}({Y_{t - p}} - \mu ) + {\theta _1}{\varepsilon _{t - 1}} + \ldots \\& + {\theta _q}{\varepsilon _{t - q}} + {\phi _s}({Y_{t - s}} - \mu ) + \ldots + {\phi _{ps}}({Y_{t - ps}} - \mu ) + {\varepsilon _t}\end{array}$$

Here, ${Y}_{t}$ represents the time series data at time *t*, *µ* is the mean of the time series, *p* is the order of the autoregressive (AR) component, ${\varphi }_{1}$, …, ${\varphi }_{p}$ are the AR coefficients, *q* is the order of the moving average (MA) component, ${\theta }_{1}$, …, ${\theta }_{q}$ are the MA coefficients, *s* is the seasonal period, ${\varphi }_{s}$, …, ${\varphi }_{ps}$ are the seasonal AR coefficients, and ${\epsilon }_{t}$ is the error term at time *t*.

The SARIMA model comprises 3 main components: (1) autoregressive (AR) component - This component captures the dependence of the current value of the time series on its past values. The order of the AR component is denoted by *p*; (2) moving average (MA) component - This component captures the dependence of the current value of the time series on past error terms. The order of the MA component is denoted by *q*; and (3) seasonal component - This component captures the periodic fluctuations in the data. The seasonal period is denoted by *s*, and the seasonal AR and MA coefficients are denoted by ${\varphi }_{s}$, …, ${\varphi }_{ps}$ and ${\theta }_{s}$, …, ${\theta }_{qs}$, respectively. Maximum likelihood estimation (MLE) can be employed to estimate the SARIMA model, and the ideal values of the model parameters can be determined by employing information criteria such as the Akaike information criterion (AIC) or Bayesian information criterion (BIC) [14].

### Data analysis

The SARIMA model was fitted using Python Grid Search [14], which automatically selected the optimal model based on the minimum AIC. The success of the model fitting was evaluated by testing the residual white noise. The model parameters were tested using MLE [13].

Data collection and collation were carried out using the Excel software (version 2021). The STL, SARIMA model, Augmented Dickey-Fuller (ADF) test, Kwiatkowski-Phillips-Schmidt-Shin (KPSS) test, Ljung-Box test, Kruskal-Wallis test, and Mann-Whitney *U* test were established using the Python software (version 3.9.13). The stationarity of the sequence was determined by checking if the *P* values of the Augmented Dickey-Fuller (ADF) test were less than the significance level (0.05) and if the autocorrelation coefficient decayed rapidly to 0. If the *P* values of the Kwiatkowski-Phillips-Schmidt-Shin (KPSS) test were less than the significance level (0.05), the sequence was considered non-stationary. The Ljung-Box test was used to check if the sequence was a white noise sequence, and if the *P* values were less than the significance level (0.05), the sequence had no randomness. If the original sequence was stationary and non-random, the model could be directly constructed. Otherwise, a d or D-order difference was applied to make the sequence stationary before constructing the model.

## Results

### Seasonal characteristics of ILI

**Fig. 1:**
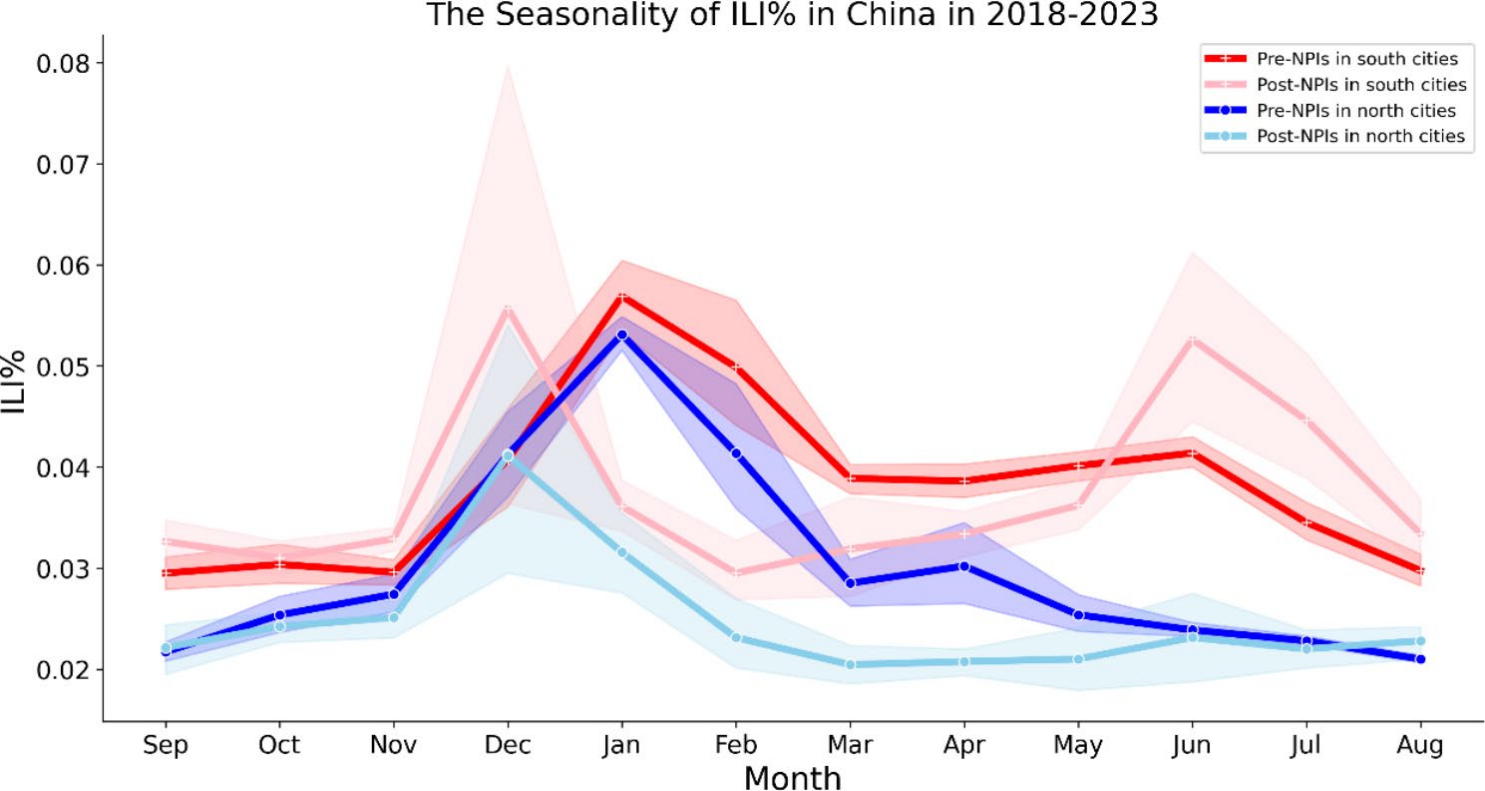
Variations observed pre- and post-implementation of NPI in cities located in North and South China. The graph displays the seasonality of the weekly percentage of patients with influenza-like illness (ILI%) in China during 2 periods: 2018 to 2019 (pre-NPIs) and 2020 to 2023 (post-NPIs). The red and light pink lines correspond to southern cities in China, while the blue and sky-blue lines correspond to northern cities. The shaded areas indicate the maximum and minimum values

The influenza data included the weekly percentage of ILI patients (ILI%). The ILI% of pre-NPIs and post-NPIs seasons were shown in Fig. 1. In general, ILI% levels were higher in southern cities than in those in North China (Fig. 1; *P* < 0.05), with peaks in winter and summer. After performing cross-correlation analysis, we discovered that both northern and southern cities in China experienced outbreaks approximately 8 weeks earlier during the NPI periods compared to the 2018–2019 season, which typically coincides with winter/summer months. The ILI% decreased significantly more during the 2020–2023 season after the implementation of NPIs than during the 2 previous seasons before NPIs (Table 1; *P* < 0.05). Additionally, infections had a much shorter duration after the implementation of NPIs (Fig. 1; Table 1), which may be linked to measures including school closures that limit viral transmission [15].

**Table 1:** Comparisons of the ILI rates in South and North China before and after the implementation of NPIs during the COVID-19 pandemic

Time series data	Kruskal-Wallis test		Mann-Whitney *U* test	
	***H-statistic***	***P***	***U-statistic***	***P***
ILI% in South cities	8.7096	0.003*	11855.5	0.003*
ILI% in North cities	29.143	< 0.001*	13524.0	< 0.001*

ILI: influenza-like illness; NPIs: non-pharmaceutical interventions

*P-value < 0.05 with statistical significance

**Fig. 2:**
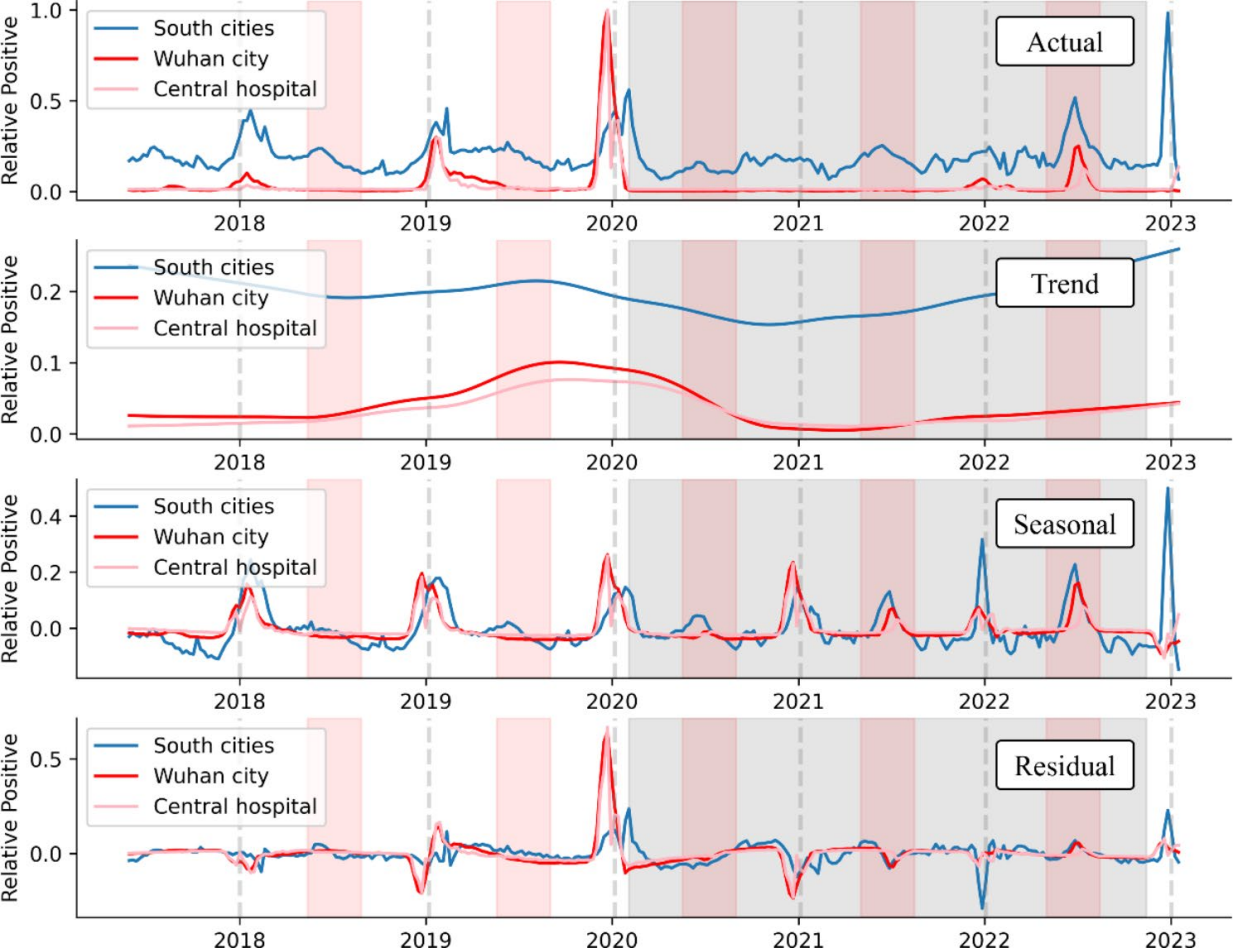
Influenza sequence characteristics in mainland China, Wuhan City, and the Central Hospital of Wuhan. The graphs display the STL analysis of the influenza data in South China (blue lines), Wuhan City (red lines), and the Central Hospital of Wuhan (light pink lines) from 2018 to 2023, including the actual data, long-term trends, seasonal trends, and residuals. The gray dashed line separates each year, while the gray shaded area indicates the period of NPIs

The ILI% time series data were further analyzed using STL (Fig. 2). The figure displays the actual data, long-term trends, seasonal trends, and residuals top to bottom. The long-term trends indicated a decrease in influenza cases under NPIs. The seasonality and periodicity of influenza with a 1-year cycle (52 weeks) were evident in the seasonal trends. The peak occurred mainly in winter and to a lesser extent in summer.

### SARIMA model

To build the SARIMA model for forecasting future influenza trends, weekly ILI% data were used. According to Table 2, all 4 of the original sequences obtained from both study areas were stationary and non-random, indicating the model could be constructed directly.

**Table 2:** Stationarity and model fitting evaluations in the original data obtained from South and North China, Wuhan City, and the Central Hospital of Wuhan

Time series data (ILI%)	ADF		KPSS		Ljung-Box	
	*t- statistic*	*P*	*χ* ^*2*^ *- statistic*	*P*	*χ* ^*2*^ *- statistic*	*P*
South Cities	-6.883	< 0.001*	0.353	0.097	290.256	< 0.001*
North Cities Wuhan City Central Hospital	-5.382 -5.662 -6.847	< 0.001* < 0.001* < 0.001*	0.259 0.172 0.161	0.100 0.100 0.100	325.231 444.401 197.877	< 0.001* < 0.001* < 0.001*

ILI: influenza-like illness; ADF: augmented Dickey-Fuller test; KPSS: Kwiatkowski-Phillips-Schmidt-Shin test

*P-value < 0.05 with statistical significance

**Fig. 3:**
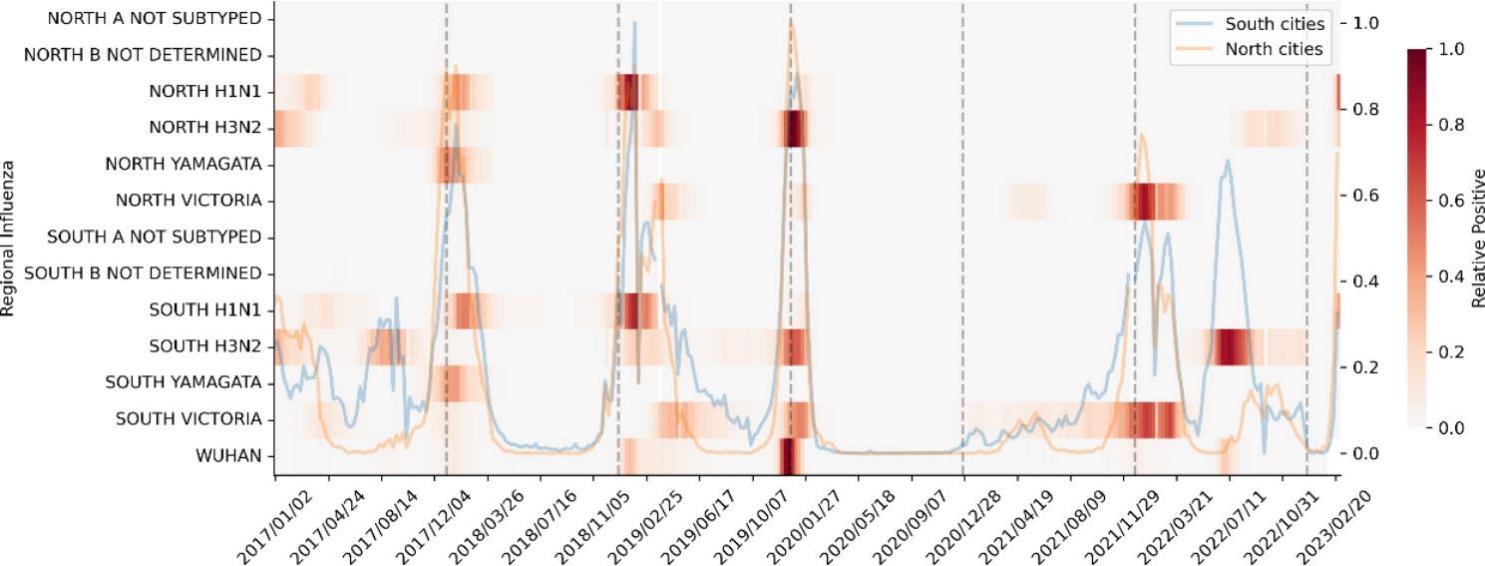
Regional and temporal distribution of influenza strains in China from 2018–2023. The heat map displays 6 types of influenza strains (H1N1, H3N2, A NOT SUBTYPED, YAMAGATA, VICTORIA, B NOT DETERMINED) in North and South China, along with influenza cases from Wuhan City. The orange and blue lines indicate North and South China, respectively, while the gray dashed lines separate each year. The *y*-axis represents the strains of the virus in different regions while the *x*-axis represents time. The dark red color represents the peak period of each strain’s outbreak during the year

As shown in Fig. 3, the low levels of ILI% during NPIs can be observed. Following the relaxation of NPIs, the first circulating strains detected were Victoria and H3N2, followed by H1N1 across mainland China.

**Fig. 4:**
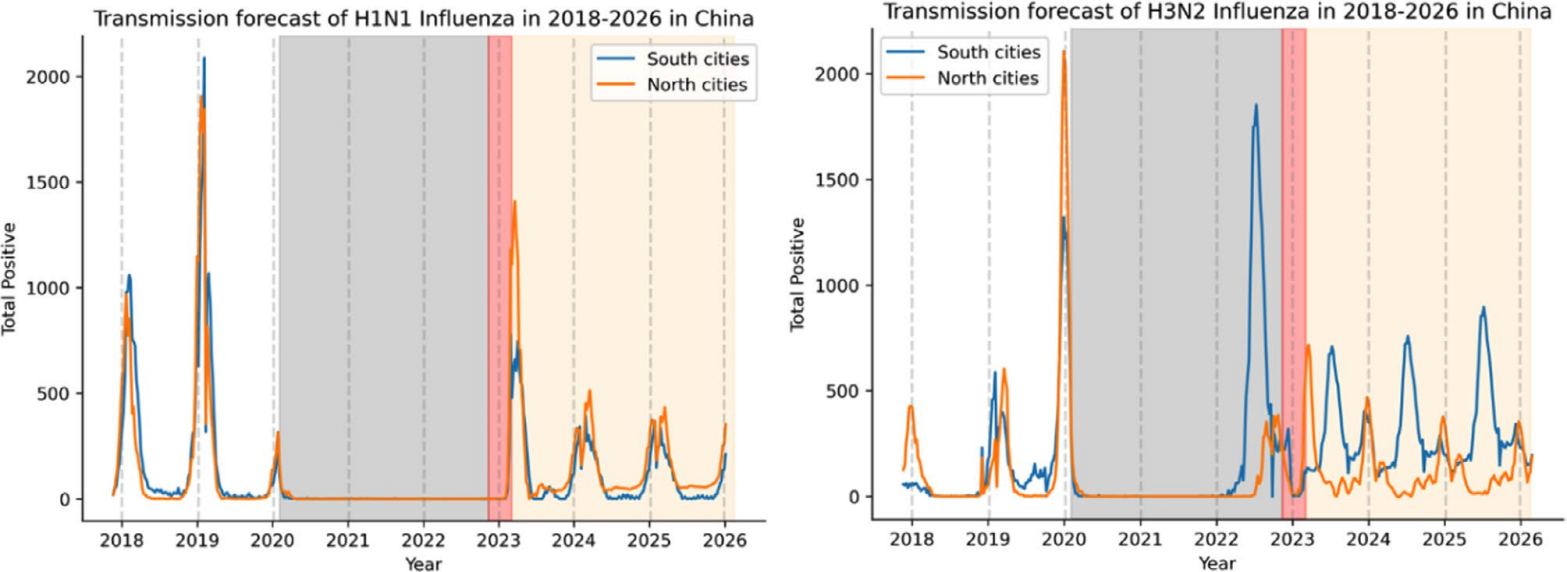
Forecasting H1N1 or H3N2 influenza trends in mainland China using the SARIMA model. The graphs display the predicted transmission of either H1N1 (*left*) or H3N2 (*right*) influenza in North or South China from 2018–2023. The blue line represents the South cities, while the orange line for the North cities. A gray dashed line is used to indicate the boundary for each year, with the gray-shaded region indicating the NPIs period, the red region representing the transitional period after NPI relaxation, and the yellow region illustrating the SARIMA model’s predicted trend for the next 3 years

**Fig. 5:**
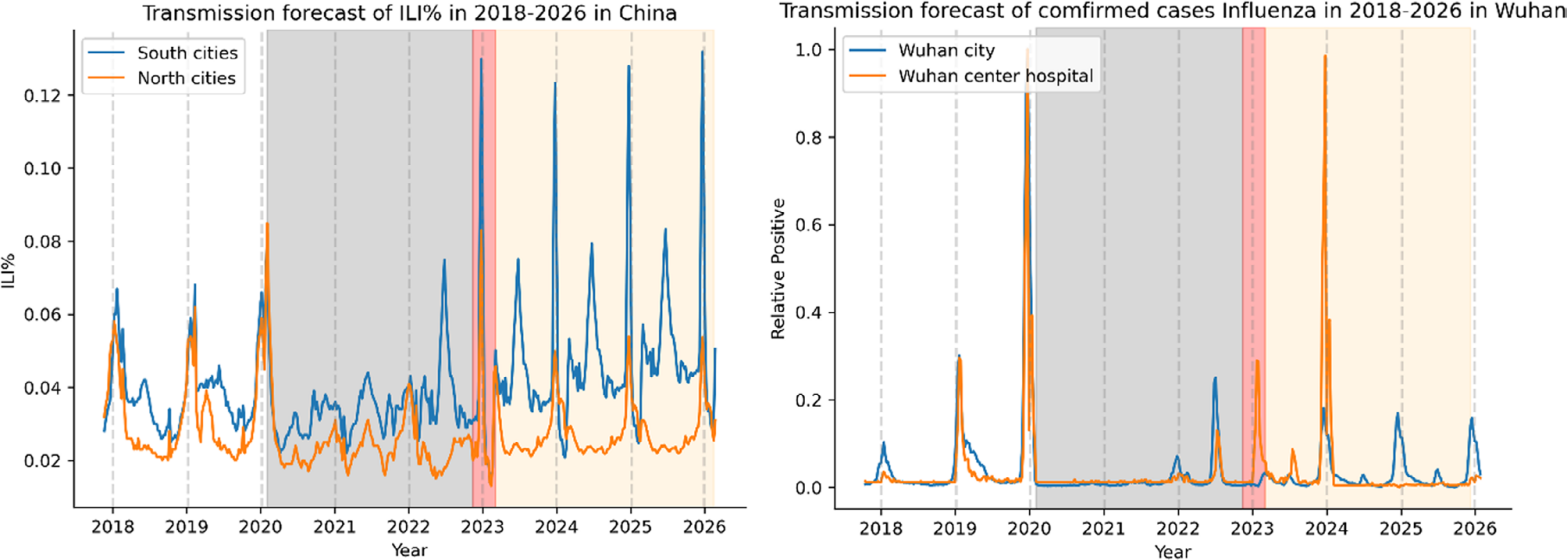
Forecasting ILI trends in mainland China and Wuhan using the SARIMA model. The graphs display the predicted transmission of ILI in mainland China (*left*) or Wuhan (*right*) from 2018–2026. The blue line corresponds to the South cities (*left*) or Wuhan City (*right*), while the orange line corresponds to the North cities (*left*) or the Central Hospital of Wuhan (*right*). A gray dashed line represents the boundary for each year, and the gray-shaded area indicates the NPIs period, while the red area represents the transitional period following the relaxation of the NPIs. The yellow area indicates the trend predicted by the SARIMA model for the next 3 years

We then utilized the SARIMA model to predict forthcoming influenza trends. According to Fig. 4, the H1N1 outbreak cycle in both North and South cities in China is synchronized while H3N2 is anticipated to occur in the summer in South cities and in the winter in North cities within the next 3 years. The ILI burden in the northern and southern cities is expected to rise over the next 3 years (Fig. 5, left). The ILI% levels in the southern cities are significantly higher throughout the year, primarily in the winter and, to a less extent, in the summer. Northern cities experience higher levels mainly during the winter (Fig. 5, left). In Wuhan City and the Central Hospital of Wuhan, influenza levels are projected to peak in the winter of 2024, with 2 smaller peaks expected during the summer of 2023 (Fig. 5, right).

## Discussion

Global surveillance data indicates that the 2022 influenza season manifests greater severity than the pre-pandemic counterpart, commencing earlier than the usual timeframe (https://www.who.int/tools/flunet; accessed 16 Aug 2023). In our research conducted throughout China, we observed that during periods of NPIs, the peak flu outbreaks in winter occurred roughly 8 weeks earlier compared to non-NPIs periods. Furthermore, these outbreaks were diminished in scale, both in terms of their duration and the total number of infections. The rationale behind these observations might involve intricate factors that interact in a mutually influential manner. Significant reduction in community exposure to influenza while NPIs are preserved aimed at the prevention of virus transmission. Long-time NPIs have reduced the levels of influenza immunity in the population that is raised through natural infection. Influenza viruses evolve under strong positive selection driven by pressure to escape from preexisting population immunity and rapidly mutate and evolve through selection, genetic drift, and reassortment [16–18]. Viral–viral interactions [19], immune debt [20], and decline in evolutionary pressure of Influenza viruses might reshape the new balance in NPIs period [21]. With the large-scale vaccination of COVID-19 [22] and the gradual liberalization of NPIs, the environment affecting the transmission and evolution of influenza viruses have changed accordingly, and consequently the time and scale of the outbreak change.

According to our findings, following the relaxation of these measures, there was a marked increase in influenza activity in China, aligning with earlier reports [23–25]. Using the time-series SARIMA model, we predicted influenza activities and examined the prevalence of major circulating strains, H1N1 and H3N2 [23–27]. In temperate regions such as Europe and North America, influenza typically results in winter epidemics [28–30]. However, tropical and subtropical regions exhibit less regular seasonality [29, 31]. In subtropical regions, influenza is present throughout the year and is not restricted to winter periods. Instead, a two-peak pattern is observed with peaks in both winter and spring/summer [32, 33]. In China, the majority of northern cities exhibit a temperate climate, while the majority of southern cities experience a subtropical climate [34]. The hot, humid, and mild environment of southern region is favourable for the influenza virus to survive throughout the year [35], while the colder and drier environment of northern region is less suitable for influenza virus survival [36]. Additionally, southern cities have a high population density and a considerable number of susceptible individuals. The variations between cities in the southern and northern regions were also identified in this study. Our results indicate that H1N1 strains were more common in mainland China during the winter season, from January to April [24]; while H3N2 strains were more prevalent in South China from May to July and in North China during the winter season. In general, ILI% levels were higher in southern cities than in northern cities, with levels concentrated in winter and summer. However, in northern cities, ILI% levels were mainly concentrated in winter. The findings in southern cities, including Wuhan City, were verified using data collected from the Central Hospital of Wuhan, influenza activity primarily peaked during the small summer peak and winter peak.

In certain countries and regions, influenza has the potential to cause a widespread outbreak, posing significant threats to public health and resulting in economic losses [11, 37], and deaths from influenza [38]. We thus recommend that local governments encourage vaccination during the transition period between summer and winter to mitigate economic losses and mortality associated with influenza.

There are several limitations in this study. We did not consider other factors that could impact influenza transmission rates, including shifts in vaccination rates, climate patterns, or population mobility. Additionally, this study did not thoroughly examine the specific NPIs that were enforced in China and their impact on influenza transmission rates. This study may also have some biases and limitations associated with the time-series method employed. One limitation is that this method relies on historical data and may not be able to accommodate sudden changes in the virus or shifts in population behavior. Moreover, the impact of interventions may not be adequately captured by these methods. It is worth noting that SARIMA may not be the most suitable method for long-term predictions, and our confidence in predictions may be restricted to short-term predictions. Lastly, time-series methods can be sensitive to outliers or anomalous events, leading to potentially inaccurate forecasts. In the future, a multi-modal approach utilizing artificial intelligence (AI)-based large-scale model can be employed to formulate an infectious disease model. This will involve gathering multi-dimensional real-world data and developing a predictive AI model specifically tailored for respiratory infectious diseases all around the world.

## Conclusion

In this study, we report that NPIs measures can effectively slow down the spread of the influenza virus and reduce the magnitude of influenza outbreaks. Over the next 3 years, influenza in South China, including Wuhan City, is anticipated to experience a minor outbreak in the summer and a peak in winter.

## Data Availability

The data that support the findings of this study are available from Xiaofan Liu upon reasonable request and with permission of The Central Hospital of Wuhan, Hubei, China.

## Abbreviations

CNIC: Chinese National Influenza Center
ILI: Influenza-like Illness
NPIs: Non-pharmaceutical Interventions
SARIMA: Seasonal Autoregressive Integrated Moving Average
SARS-CoV-2: Severe Acute Respiratory Syndrome Coronavirus 2
STL: Seasonal-trend Decomposition using Loess
