# Correction: Impact of non-pharmaceutical interventions during COVID-19 on future influenza trends in Mainland China

**DOI:** 10.1186/s12879-024-09577-6

**Published:** 2024-07-04

**Authors:** Xiaofan Liu, Ying Peng, Zhe Chen, Fangfang Jiang, Fang Ni, Zhiyong Tang, Xun Yang, Cheng Song, Mingli Yuan, Zhaowu Tao, Junjie Xu, Ying Wang, Qiong Qian, Rob M. Ewing, Ping Yin, Yi Hu, Weihua Wang, Yihua Wang

**Affiliations:** 1 Department of Pulmonary and Critical Care Medicine, The Central Hospital of Wuhan, Tongji Medical College, Huazhong University of Science and Technology, Wuhan, Hubei 430014 China; 2 Wuhan Centers for Disease Control and Prevention, Wuhan, Hubei 430024 China; 3 Biological Sciences, Faculty of Environmental and Life Sciences, University of Southampton, Southampton, SO17 1BJ UK; 4 Department of Biostatistics, University of Iowa, Iowa City, IA 52242 USA; 5 Institute for Life Sciences, University of Southampton, Southampton, SO17 1BJ UK; 6 Department of Epidemiology and Biostatistics, School of Public Health, Tongji Medical College, Huazhong University of Science and Technology, Wuhan, Hubei 430014 China; 7 NIHR Southampton Biomedical Research Centre, University Hospital Southampton, Southampton, SO16 6YD UK

Following publication of the original article [1], the authors note that the affiliation 1 “Department of Pulmonary and Critical Care Medicine, Tongji Medical College, The Central Hospital of Wuhan, Huazhong University of Science and Technology, Wuhan, 430014, Hubei, China” should instead read “Department of Pulmonary and Critical Care Medicine, The Central Hospital of Wuhan, Tongji Medical College, Huazhong University of Science and Technology, Wuhan 430014, Hubei, China”.
